# Crystal structure, Hirshfeld analysis and a mol­ecular docking study of a new inhibitor of the Hepatitis B virus (HBV): ethyl 5-methyl-1,1-dioxo-2-{[5-(pentan-3-yl)-1,2,4-oxa­diazol-3-yl]meth­yl}-2*H*-1,2,6-thia­diazine-4-carboxyl­ate

**DOI:** 10.1107/S2056989019015986

**Published:** 2020-01-01

**Authors:** Alexandre V. Ivachtchenko, Sergiy M. Kovalenko, Dmitry V. Kravchenko, Oleg D. Mitkin, Vladimir V. Ivanov, Thierry Langer

**Affiliations:** a ChemRar Research and Development Institute, 7 Nobel St, Innovation Center, Skolkovo Territory, Moscow, 143026, Russian Federation; b V.N. Karazin Kharkiv National University, 4 Svobody Sq., Kharkiv, 61077, Ukraine; c Chemical Diversity Research Institute, 2A Rabochaya St, Khimki, Moscow Region, 141400, Russian Federation; d University of Vienna, Althanstrasse 14, A-1090, Vienna, Austria

**Keywords:** crystal structure, 2*H*-1,2,6-thia­diazine 1,1-dioxide, hepatitis B, HBV, hydrogen bonding, Hirshfeld surface analysis, mol­ecular docking study

## Abstract

The title compound, C_15_H_22_N_4_O_5_S, was prepared *via* alkyl­ation of 3-(chloro­meth­yl)-5-(pentan-3-yl)-1,2,4-oxa­diazole in anhydrous dioxane in the presence of tri­ethyl­amine. The thia­diazine ring has an envelope conformation with the S atom displaced by 0.4883 (6) Å from the mean plane through the other five atoms. The planar 1,2,4-oxa­diazole ring is inclined to the mean plane of the thia­diazine ring by 77.45 (11)°. In the crystal, mol­ecules are linked by C—H⋯N hydrogen bonds, forming chains propagating along the *b-*axis direction. Hirshfeld surface analysis and two-dimensional fingerprint plots have been used to analyse the inter­molecular contacts present in the crystal. Mol­ecular docking studies were use to evaluate the title compound as a potential system that inter­acts effectively with the capsid of the Hepatitis B virus (HBV), supported by an experimental *in vitro* HBV replication model.

## Abstract 2

The title compound, a new inhibitor of the Hepatitis B virus (HBV), was prepared *via* alkyl­ation of 3-(chloro­meth­yl)-5-(pentan-3-yl)-1,2,4-oxa­diazole in anhydrous dioxane in the presence of tri­ethyl­amine.

## Chemical context   

Derivatives of 2*H*-1,2,6-thia­diazine 1,1-dioxide demonstrate anti­viral (Martínez *et al.*, 1999 ▸; Esteban *et al.*, 1997 ▸, 1995 ▸), cannabinoid (Cano *et al.*, 2007 ▸), anti­diabetic (Goyal & Bhargava, 1989 ▸; Jain & Malik, 1983 ▸), anti-HIV-1 (Breining *et al.*, 1995 ▸) and anti­parasitic (Arán *et al.*, 1986 ▸) activities. In addition, such derivatives are patent protected as pain relievers and anti­pyretic drugs (Giraldez *et al.*, 1989 ▸). Heterocyclic homologues of 2*H*-1,2,6-thia­diazine-1,1-dioxides are inhibitors of human cytomegalovirus (Martínez *et al.*, 2003 ▸), Cruzi triposome (Álvarez *et al.*, 2010 ▸) and diuretics (Goya *et al.*, 1992 ▸). In a continuation of our efforts to obtain new HBV inhibitors for the treatment and prevention of human HBV infections (Ivachtchenko *et al.*, 2019 ▸; Ivashchenko *et al.*, 2019 ▸; Kovalenko *et al.*, 2019 ▸), we initiated the design, synthesis, and anti-hepatitis B virus activity testing of the new 2*H*-1,2,6-thia­diazine 1,1-dioxide derivative, ethyl 5-methyl-1,1-dioxo-2-{[5-(pentan-3-yl)-1,2,4-oxa­diazol-3-yl]meth­yl}-2*H*-1,2,6-thia­diazine-4-carb­ox­yl­ate (**3**).

**Figure 1:**
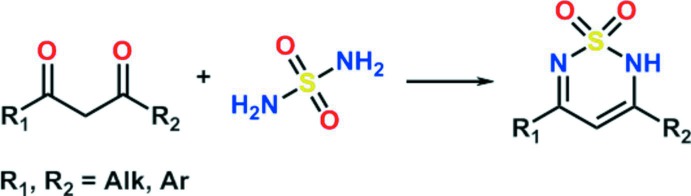
Synthesis of 2*H*-1,2,6-thia­diazine 1,1-dioxide *via* inter­molecular cyclization of sulfamide with the corresponding 1,3-diketone.

**Figure 2:**
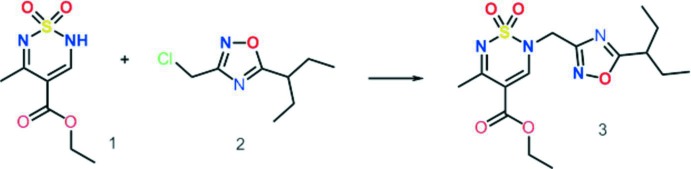
Synthesis of the title compound **3**.

One of the main methods of 2*H*-1,2,6-thia­diazine 1,1-dioxide synthesis is the inter­molecular cyclization of sulfamide with the corresponding 1,3-diketone (Cheone, 2001 ▸; Alberola *et al.*, 1991 ▸), as shown in Fig. 1 ▸. The synthesis of the title compound (**3**) is illustrated in Fig. 2 ▸. The starting product **1** was converted to compound **3** by alkyl­ation of 3-(chloro­meth­yl)-5-(pentan-3-yl)-1,2,4-oxa­diazole (**2)** in anhydrous dioxane in the presence of tri­ethyl­amine.

Single-crystal X-ray diffraction analysis and different spectroscopic techniques confirm the assigned chemical structure of the title compound. Mol­ecular docking simulations were also carried out.
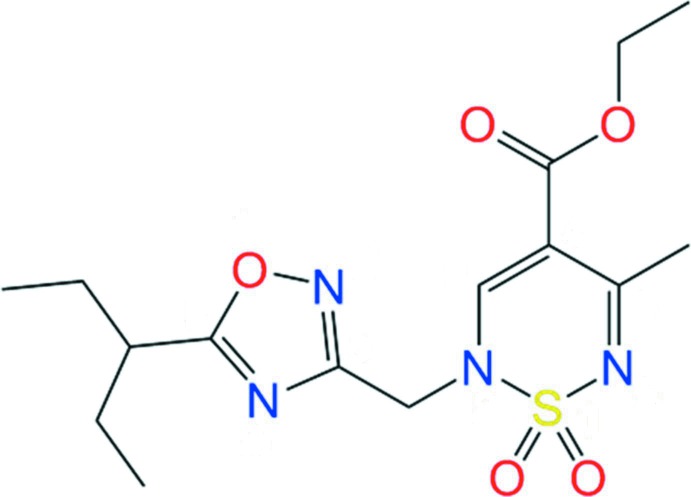


## Structural commentary   

**Figure 3:**
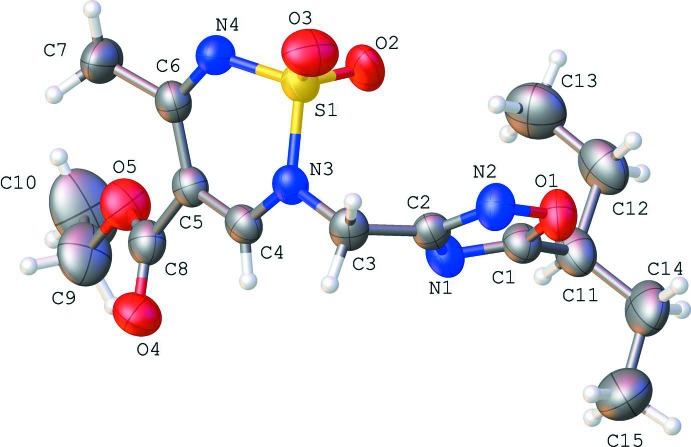
The mol­ecular structure of compound **3**, with atom labelling. Displacement ellipsoids are drawn at the 50% probability level.

The mol­ecular structure of compound **3**, is illustrated in Fig. 3 ▸. The thia­dazine ring (S1/N3/N4/C4–C6) has an envelope conformation [puckering parameters: amplitude *Q* = 0.3314 (17) Å, θ = 114.2 (3)°, φ = 182.5 (4)°], with atom S1 displaced by 0.4883 (6) Å from the mean plane through the other five atoms. The planar 1,2,4-oxa­diazole ring (O1/N1/N2/C1/C2; r.m.s. deviation = 0.008 Å) is inclined to the mean plane of the thia­diazine ring by 77.45 (11)°. The oxa­diazole ring is almost normal to the C4—N3 endocyclic bond, with the C4—N3—C3—C2 torsion angle being 92.4 (3)°, and it is twisted with respect to the N3-C3 exocyclic bond, with the N3—C3—C2—N2 torsion angle being 127.1 (2)°. The ester substituent is not completely planar and it is twisted in relation to the C4—C5 endocyclic bond; the C4—C5—C8—O4 torsion angle is 23.7 (4)° as a result of steric repulsion between the hydrogen atom of the thia­diazine-dioxide ring and the oxygen atom of the ester substituent. The *iso*-pentyl group has an all-*trans* conformation [the C13—C12—C11—C14 and C12—C11—C14—C15 torsion angles are 173.4 (3) and −175.5 (3)°, respectively] and is oriented in such a way that the N1—C1—C11—H11 torsion angle is −6.7°. The ethyl groups of this substituent have -*sc* and +*sc*-conformations in relation to the C1—C11 bond [C1—C11—C12—C13 = −62.4 (3)° and C1—C11—C14—C15 = 61.6 (4)°].

## Supra­molecular features   

**Figure 4:**
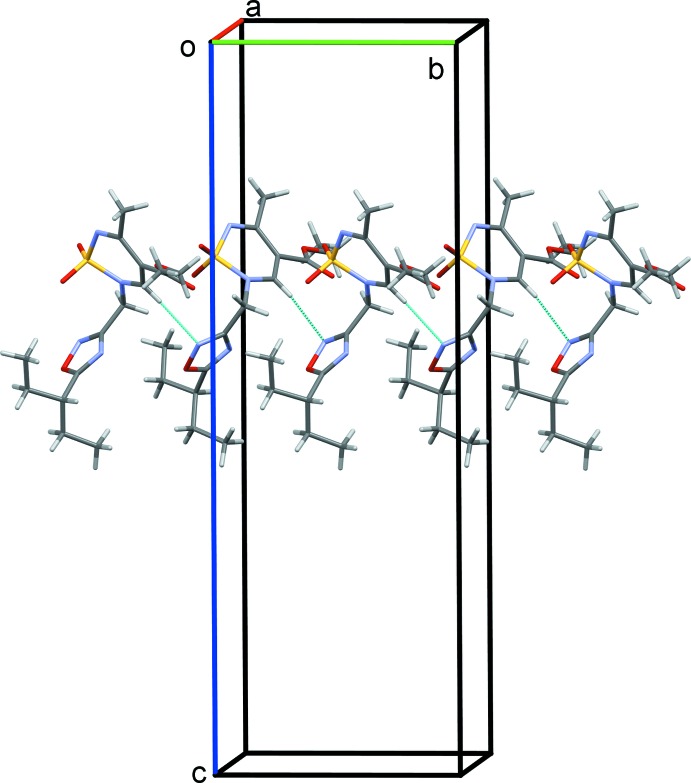
A partial view along the *a* axis of the crystal packing of compound **3**. Hydrogen bonds (Table 1 ▸) are shown as dashed lines.

In the crystal, mol­ecules are linked by C—H⋯N hydrogen bonds, forming chains propagating along the *b*-axis direction (Table 1 ▸ and Fig. 4 ▸). There are no other significant inter­molecular inter­actions present in the crystal.

## Database survey   

A search of the Cambridge Structural Database (CSD, Version 5.40, August 2019; Groom *et al.*, 2016 ▸) for the 1,2,6-thia­diazine 1,1-dioxide skeleton yielded 37 hits. Only one structure involves a carboxyl­ate in position 4, *viz*. methyl 2,3-dimethyl-5-(tri­chloro­meth­yl)-2*H*-1,2,6-thia­diazine-4-carb­oxy­l­ate-1,1-dioxide (CSD refcode ZECWAI; Onys’ko *et al.*, 2017 ▸). The thia­diazine ring has the usual envelope conformation with the S atom displaced by 0.679 (1) Å from the mean plane through the other five atoms [*cf.* 0.488 (1) Å in the title compound]. The acetate group is inclined to this mean plane by 51.3 (2)° compared to 28.98 (18)° in the title compound.

## Hirshfeld surface analysis   

**Figure 5:**
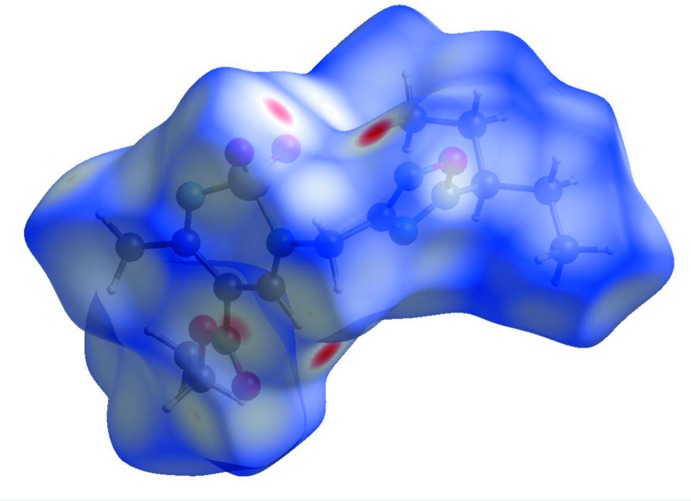
The Hirshfeld surface of compound **3**, mapped over *d*_norm_, with a fixed colour scale of −0.484 to 1.652 a.u.

The Hirshfeld surface analysis (Spackman & Jayatilaka, 2009 ▸) and the associated two-dimensional fingerprint plots (McKinnon *et al.*, 2007 ▸) were performed with *CrystalExplorer17* (Turner *et al.*, 2017 ▸). The mol­ecular Hirshfeld surfaces were obtained using a standard (high) surface resolution with the three-dimensional *d*_norm_ surface (Fig. 5 ▸), mapped over a fixed colour scale of −0.484 (red) to 1.652 (blue). There are four red spots in the *d*_norm_ surface indicating the regions of donor–acceptor inter­actions or short contacts. A list of short contacts in the crystal of compound **3** are given in Table 2 ▸.

**Figure 6:**
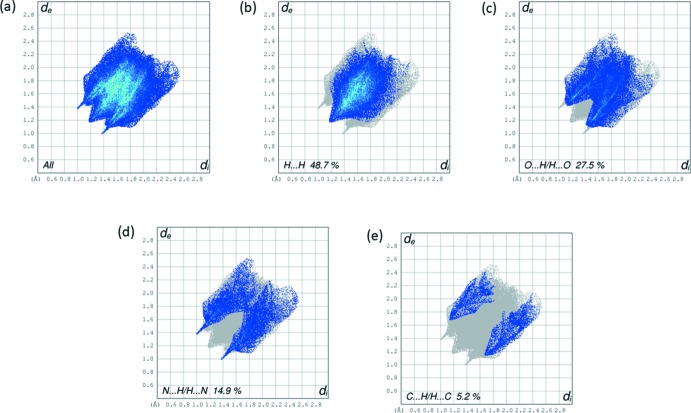
(*a*) The two-dimensional fingerprint plot for compound **3**, and delineated into (*b*) H⋯H (48.7%), (*c*) O⋯H/H⋯O (27.5%), (*d*) N⋯H/H⋯N (14.9%) and (*e*) C⋯H/H⋯C (5.2%) contacts.

The inter­molecular inter­actions in the crystal of the title compound are shown on the two-dimensional fingerprint plots presented in Fig. 6 ▸. The contribution of the O⋯H/H⋯O contacts, corresponding to the C—H⋯O inter­actions, is represented by a pair of sharp spikes. The inter­actions appear in the middle of the scattered points in the two-dimensional fingerprint plot with a contribution to the overall Hirshfeld surface of 27.5% (Fig. 6 ▸ *c*). The fingerprint plots indicate that the principal contributions are from H⋯H (48.7%; Fig. 6 ▸ *b*), O⋯H/H⋯O (27.5%; Fig. 6 ▸ *c*), N⋯H/H⋯N (14.9%; Fig. 6 ▸ *d*) and C⋯H/H⋯C (5.2%; Fig. 6 ▸ *e*) contacts.

## Mol­ecular docking evaluation   

The title mol­ecule (**3**) was investigated as a potential system that can inter­act effectively with the capsid of the Hepatitis B virus (HBV). We performed mol­ecular modelling of the inter­action of title mol­ecule with core HBV proteins including 5E0I, 5GMZ, 5WRE and 5T2P. The crystal structures of these proteins were obtained at high resolution (1.5–2 Å), all necessary information about the crystal structures being downloaded from the Protein Data bank (Berman *et al.*, 2000 ▸; accessed on 24 July 2019). The pharmacophore model was generated by using the *Ligandscout 4.3* program (Wolber & Langer, 2005 ▸; accessed on 24 July 2019). All of the above-mentioned protein structures contain six chains (designated as *A*, *B*, *C*, *D*, *E*, *F*). The docking poses of ligands (reference mol­ecules) were extracted from the obtained crystallographic data. For the correct choice of appropriate chain and poses for mol­ecular modelling (docking), all the reference ligands were re-docked. According to our calculations, the minimal values of the residual mean-square deviations (r.m.s.d.) for the geometry were obtained for poses in the *D* chains (r.m.s.d. < 1 Å). Hence, the active-site selection and corresponding pharmacophore analyses were performed for the *D* chains of the above-mentioned proteins. The most significant information is collected in Table 3 ▸.

**Figure 7:**
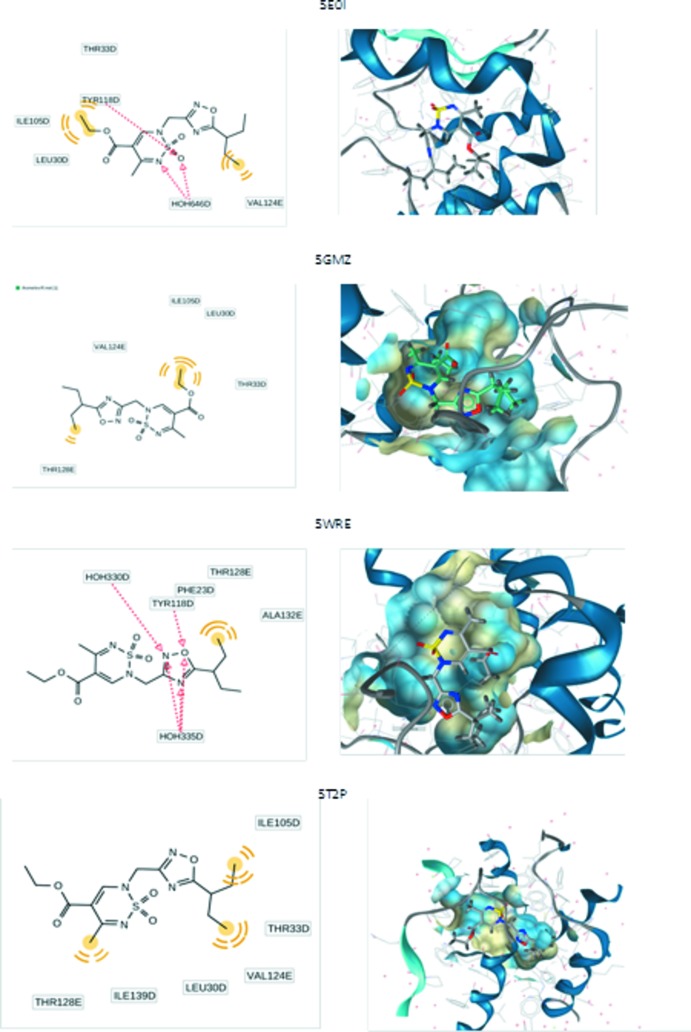
Calculated docking poses for the complex ‘title mol­ecule–protein’.

As can be seen from Table 3 ▸, our system demonstrated rather large values of binding affinity for all the proteins. The corresponding graphical representation describes the pharmacophore environment of the ligands (Fig. 7 ▸, left) and poses in proteins (Fig. 7 ▸, right). The red lines designate hydrogen-bond acceptors, while yellow lines designated hydro­phobic inter­actions.

**Figure 8:**
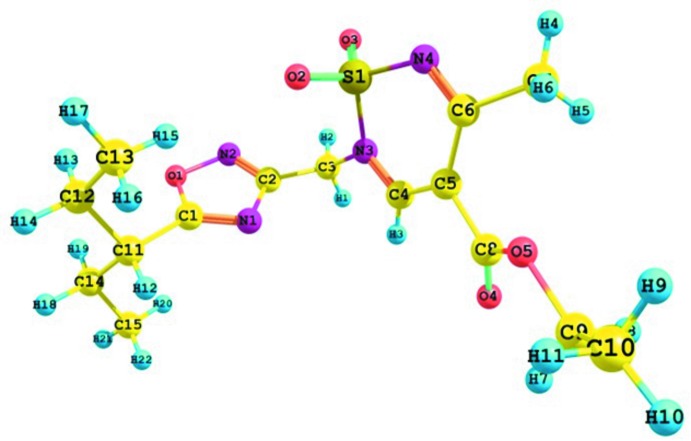
Calculated structure of the title compound.

It should be noted that the geometrical configuration of the title mol­ecule (as ligand immersed to protein) essentially depends on the pharmacophore surroundings. The most significant geometrical parameters (torsion angles) obtained from the docking procedure are compared with results of the non-empirical calculations and X-ray data in Table 4 ▸. The calculated structure of the title compound is illustrated in Fig. 8 ▸. The *ab initio* calculations were performed by using density functional theory with M062x functional and cc-pVDZ basis set.

The obtained data demonstrate significant geometrical relaxation associated with immersion of the mol­ecule in a protein.

## *in vitro* HBV replication model   

The biological activity of the title compound **3** was studied using an experimental *in vitro* hepatitis B virus infection model maintaining a full virus replication cycle. This model based on the human hepatoma line HepG2 stably transfected with the NTCP gene (Sun *et al.*, 2016 ▸) was developed in our laboratories for identification of viral entry inhibitors able to prevent development of resistant HBV forms (Ivachtchenko *et al.*, 2019*b* ▸). Compound **3** demonstrated 80% inhibition of HBV replication (in 10 µ*M* concentration) in this model and could be considered to be a promising candidate for the development of a potent anti-HBV medicine capable of preventing the development of resistant HBV forms (Donkers *et al.*, 2017 ▸).

## Synthesis and crystallization   

The synthesis of the title compound is illustrated in Fig. 2 ▸. 3-(Chloro­meth­yl)-5-(pentan-3-yl)-1,2,4-oxa­diazole (**2**) (1.1 mmol, 208 mg) was added to a solution of ethyl 5-methyl-2*H*-1,2,6-thia­diazine-4-carboxyl­ate 1,1-dioxide (**1**) (1.0 mmol, 218 mg) and NEt_3_ (1.1 mmol) in 1 ml of DXN (2,6-dimethyl-1,3-dioxan-4-yl acetate) and the resulting mixture was heated at 353 K for 12 h. After cooling to room temperature, the solution was diluted with water (50 ml) and extracted with CH_2_Cl_2_, dried over MgSO_4_, filtered and concentrated *in vacuo*. The product, compound **3**, was purified by crystallization from aceto­nitrile giving a white crystalline powder (yield 308 mg, 83%; m.p. 338–339 K). Further crystallization by slow evaporation of an aceto­nitrile solution yielded colourless irregularly shaped crystals.

## Refinement   

Crystal data, data collection and structure refinement details are summarized in Table 5 ▸. H atoms were included in calculated positions and treated as riding on their parent C atom: C—H = 0.93–0.98 Å with *U*_iso_(H) = 1.5*U*_eq_(C-meth­yl) and 1.2*U*_eq_(C) for other H atoms.

## Supplementary Material

Crystal structure: contains datablock(s) Global, I. DOI: 10.1107/S2056989019015986/su5529sup1.cif

Structure factors: contains datablock(s) I. DOI: 10.1107/S2056989019015986/su5529Isup2.hkl

Supporting information file. DOI: 10.1107/S2056989019015986/su5529Isup3.cml

CCDC reference: 1968398

Additional supporting information:  crystallographic information; 3D view; checkCIF report

## Figures and Tables

**Table 1:** Hydrogen-bond geometry (Å, °)

*D*—H⋯*A*	*D*—H	H⋯*A*	*D*⋯*A*	*D*—H⋯*A*
C4—H4⋯N2^i^	0.93	2.54	3.431 (3)	161

Symmetry code: (i) 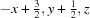.

**Table 2:** Short contacts (Å) in the crystal of compound **3**

Atom1⋯Atom2	Length	Length – vdW
S1.·H7*B* ^i^	3.090	0.090
O2⋯H7*B* ^i^	2.813	0.093
H7*A*⋯O4^i^	2.687	−0.033
H12*B*⋯N2^ii^	2.772	0.022
O4⋯H14*A* ^iii^	2.700	−0.020
C8⋯H14*A* ^iii^	2.913	0.013
C6⋯H10*B* ^iv^	2.978	0.078
O2⋯C3^v^	3.275	0.055
O2⋯H3*A* ^v^	2.634	−0.086
O3⋯C4^v^	3.077	−0.143
N2⋯H3*A* ^v^	2.816	0.066
N2⋯H*A* ^v^	2.538	−0.212
H3*B*⋯O4^v^	2.799	0.079

Symmetry codes: (i) −*x* + 1, *y* − 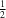, −*z* + 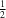; (ii) *x* − 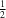, −*y* + 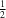, −*z* + 1; (iii) −*x* + 1, −*y* + 1, −*z* + 1; (iv) −*x* + 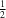, *y* − 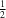, *z*; (v) −*x* + 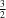, *y* − 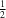, *z*.

**Table 3:** Binding affinity parameters of title mol­ecule with HBV core proteins

PDB refcode	Est. binding energy (kcal mol^−1^)	Binding affinity score
5E0I	−14.54	−25.2
5GMZ	−14.30	−14.99
5WRE	−16.03	−8.28
5T2P	−17.05	−22.34

**Table 4:** Torsion angles (°) comparison for X-ray, *ab *initio** and docking data

Torsion angle	X-ray	M062x/cc-pVDZ	5E0I	5GMZ	5WRE	5T2P
O2—S1—N4—C6	83.5 (2)	78.7	93.13	93.2	93.1	137.4
C5—C8—O5—C9	178.4 (2)	178.9	−112.9	−79.3	−110.4	−163.1
S1—N3—C3—C2	−71.7 (2)	−71.5	−148.5	−126.7	−172.5	−80
O1—C1—C11—C14	57.8 (3)	49.9	50.1	−112.9	9.8	52.8

**Table 5:** Experimental details

Crystal data	
Chemical formula	C_15_H_22_N_4_O_5_S
*M*_r_	370.42
Crystal system, space group	Orthorhombic, *P* *b* *c* *a*
Temperature (K)	293
*a*, *b*, *c* (Å)	12.4962 (5), 9.9237 (4), 29.5925 (15)
*V* (Å^3^)	3669.7 (3)
*Z*	8
Radiation type	Mo *K*α
μ (mm^−1^)	0.21
Crystal size (mm)	0.4 × 0.2 × 0.1

Data collection	
Diffractometer	Rigaku Oxford Diffraction Xcalibur, Sapphire3
Absorption correction	Multi-scan (*CrysAlis PRO*; Rigaku OD, 2018 ▸)
*T*_min_, *T*_max_	0.466, 1.000
No. of measured, independent and observed [*I* > 2σ(*I*)] reflections	26547, 3211, 2510
*R*_int_	0.070
(sin θ/λ)_max_ (Å^−1^)	0.595

Refinement	
*R*[*F* ^2^ > 2σ(*F* ^2^)], *wR*(*F* ^2^), *S*	0.050, 0.127, 1.06
No. of reflections	3211
No. of parameters	230
H-atom treatment	H-atom parameters constrained
Δρ_max_, Δρ_min_ (e Å^−3^)	0.24, −0.32

Computer programs: *CrysAlis PRO* (Rigaku OD, 2018 ▸), *SHELXT* (Sheldrick, 2015*a* ▸), *SHELXL* (Sheldrick, 2015*b* ▸), *OLEX2* (Dolomanov *et al.*, 2009 ▸), *Mercury* (Macrae *et al.*, 2008 ▸), *PLATON* (Spek, 2009 ▸) and *publCIF* (Westrip, 2010 ▸).

## supplementary crystallographic information

### Crystal data

**Table:**

C_15_H_22_N_4_O_5_S	*D*_x_ = 1.341 Mg m^−^^3^
*M**_r_* = 370.42	Mo *K*α radiation, λ = 0.71073 Å
Orthorhombic, *P**b**c**a*	Cell parameters from 3271 reflections
*a* = 12.4962 (5) Å	θ = 3.6–23.3°
*b* = 9.9237 (4) Å	µ = 0.21 mm^−^^1^
*c* = 29.5925 (15) Å	*T* = 293 K
*V* = 3669.7 (3) Å^3^	Block, colourless
*Z* = 8	0.4 × 0.2 × 0.1 mm
*F*(000) = 1568	

### Data collection

**Table:**

Rigaku Oxford Diffraction Xcalibur, Sapphire3 diffractometer	3211 independent reflections
Radiation source: fine-focus sealed X-ray tube, Enhance (Mo) X-ray Source	2510 reflections with *I* > 2σ(*I*)
Graphite monochromator	*R*_int_ = 0.070
Detector resolution: 16.1827 pixels mm^-1^	θ_max_ = 25.0°, θ_min_ = 3.0°
ω scans	*h* = −14→14
Absorption correction: multi-scan (CrysAlis PRO; Rigaku OD, 2018)	*k* = −11→11
*T*_min_ = 0.466, *T*_max_ = 1.000	*l* = −25→35
26547 measured reflections	

### Refinement

**Table:**

Refinement on *F*^2^	Primary atom site location: dual
Least-squares matrix: full	Secondary atom site location: difference Fourier map
*R*[*F*^2^ > 2σ(*F*^2^)] = 0.050	Hydrogen site location: difference Fourier map
*wR*(*F*^2^) = 0.127	H-atom parameters constrained
*S* = 1.06	*w* = 1/[σ^2^(*F*_o_^2^) + (0.053*P*)^2^ + 1.1666*P*] where *P* = (*F*_o_^2^ + 2*F*_c_^2^)/3
3211 reflections	(Δ/σ)_max_ < 0.001
230 parameters	Δρ_max_ = 0.24 e Å^−^^3^
0 restraints	Δρ_min_ = −0.32 e Å^−^^3^

### Special details

**Table:**

Geometry. All esds (except the esd in the dihedral angle between two l.s. planes) are estimated using the full covariance matrix. The cell esds are taken into account individually in the estimation of esds in distances, angles and torsion angles; correlations between esds in cell parameters are only used when they are defined by crystal symmetry. An approximate (isotropic) treatment of cell esds is used for estimating esds involving l.s. planes.

### Fractional atomic coordinates and isotropic or equivalent isotropic displacement parameters (Å^2^)

**Table:**

	*x*	*y*	*z*	*U*_iso_*/*U*_eq_	
S1	0.66349 (5)	0.40561 (6)	0.31677 (2)	0.0462 (2)	
O1	0.74895 (14)	0.29562 (18)	0.46836 (6)	0.0577 (5)	
O2	0.60666 (16)	0.31342 (17)	0.34444 (7)	0.0646 (6)	
O3	0.76374 (15)	0.3626 (2)	0.30037 (8)	0.0726 (6)	
O4	0.47430 (16)	0.86020 (18)	0.34934 (7)	0.0668 (6)	
O5	0.35185 (14)	0.7296 (2)	0.31536 (7)	0.0654 (6)	
N1	0.65072 (15)	0.4628 (2)	0.44365 (7)	0.0472 (5)	
N2	0.80748 (16)	0.3561 (2)	0.43291 (8)	0.0514 (5)	
N3	0.68614 (14)	0.54419 (19)	0.34784 (7)	0.0415 (5)	
N4	0.58953 (16)	0.4585 (2)	0.27686 (7)	0.0495 (5)	
C1	0.65689 (19)	0.3647 (2)	0.47179 (9)	0.0463 (6)	
C2	0.74512 (17)	0.4525 (2)	0.42013 (8)	0.0402 (5)	
C3	0.77420 (18)	0.5405 (2)	0.38103 (9)	0.0467 (6)	
H3A	0.788916	0.631017	0.391704	0.056*	
H3B	0.838461	0.506142	0.366693	0.056*	
C4	0.61014 (18)	0.6400 (2)	0.34937 (8)	0.0427 (6)	
H4	0.615584	0.705875	0.371592	0.051*	
C5	0.52574 (18)	0.6464 (2)	0.32039 (8)	0.0412 (6)	
C6	0.52217 (19)	0.5588 (2)	0.28224 (9)	0.0457 (6)	
C7	0.4479 (2)	0.5814 (3)	0.24327 (10)	0.0660 (8)	
H7A	0.471161	0.528646	0.217909	0.099*	
H7B	0.448204	0.675101	0.235238	0.099*	
H7C	0.376781	0.554846	0.251685	0.099*	
C8	0.4500 (2)	0.7587 (3)	0.32946 (9)	0.0497 (6)	
C9	0.2702 (2)	0.8316 (4)	0.32370 (14)	0.0881 (11)	
H9A	0.275933	0.864784	0.354432	0.106*	
H9B	0.279751	0.906902	0.303192	0.106*	
C10	0.1671 (3)	0.7707 (5)	0.31670 (19)	0.1275 (19)	
H10A	0.163950	0.732718	0.286891	0.191*	
H10B	0.112282	0.837879	0.319856	0.191*	
H10C	0.156092	0.700900	0.338674	0.191*	
C11	0.5779 (2)	0.3135 (3)	0.50547 (10)	0.0601 (7)	
H11	0.517153	0.375927	0.506023	0.072*	
C12	0.5362 (3)	0.1749 (3)	0.49009 (12)	0.0756 (9)	
H12A	0.596527	0.113804	0.487578	0.091*	
H12B	0.489029	0.139689	0.513284	0.091*	
C13	0.4770 (3)	0.1756 (4)	0.44585 (14)	0.0939 (12)	
H13A	0.522406	0.211801	0.422662	0.141*	
H13B	0.413982	0.230344	0.448575	0.141*	
H13C	0.456836	0.085211	0.438077	0.141*	
C14	0.6254 (3)	0.3084 (3)	0.55267 (11)	0.0799 (10)	
H14A	0.572775	0.269913	0.573035	0.096*	
H14B	0.686816	0.248710	0.552328	0.096*	
C15	0.6597 (3)	0.4429 (4)	0.57096 (13)	0.1016 (13)	
H15A	0.714439	0.480074	0.551891	0.152*	
H15B	0.687317	0.431690	0.601003	0.152*	
H15C	0.599454	0.502798	0.571657	0.152*	

### Atomic displacement parameters (Å^2^)

**Table:**

	*U*^11^	*U*^22^	*U*^33^	*U*^12^	*U*^13^	*U*^23^
S1	0.0472 (4)	0.0396 (4)	0.0518 (4)	0.0034 (3)	−0.0079 (3)	0.0008 (3)
O1	0.0566 (11)	0.0612 (11)	0.0553 (12)	0.0133 (9)	0.0089 (9)	0.0161 (9)
O2	0.0825 (14)	0.0438 (10)	0.0674 (13)	−0.0180 (10)	−0.0177 (11)	0.0143 (9)
O3	0.0531 (11)	0.0764 (13)	0.0881 (16)	0.0224 (10)	−0.0045 (11)	−0.0190 (12)
O4	0.0760 (13)	0.0518 (11)	0.0725 (15)	0.0144 (10)	0.0007 (11)	−0.0081 (10)
O5	0.0439 (10)	0.0727 (13)	0.0796 (15)	0.0152 (9)	0.0022 (9)	−0.0008 (10)
N1	0.0432 (11)	0.0428 (11)	0.0555 (14)	0.0035 (9)	0.0051 (10)	0.0075 (10)
N2	0.0458 (11)	0.0587 (13)	0.0497 (14)	0.0063 (10)	0.0053 (10)	0.0090 (10)
N3	0.0394 (10)	0.0387 (11)	0.0463 (12)	−0.0029 (9)	−0.0046 (9)	0.0043 (9)
N4	0.0549 (12)	0.0466 (12)	0.0470 (13)	0.0060 (10)	−0.0082 (10)	−0.0014 (9)
C1	0.0476 (14)	0.0438 (14)	0.0474 (16)	0.0064 (11)	0.0042 (11)	0.0003 (11)
C2	0.0353 (12)	0.0417 (12)	0.0435 (14)	−0.0036 (10)	−0.0049 (10)	−0.0006 (10)
C3	0.0374 (12)	0.0491 (14)	0.0536 (16)	−0.0082 (11)	−0.0061 (11)	0.0061 (12)
C4	0.0438 (13)	0.0377 (12)	0.0466 (15)	−0.0035 (11)	0.0052 (11)	0.0043 (10)
C5	0.0372 (12)	0.0410 (13)	0.0453 (15)	0.0003 (10)	0.0036 (10)	0.0056 (10)
C6	0.0454 (13)	0.0421 (13)	0.0496 (16)	−0.0006 (11)	−0.0025 (11)	0.0073 (11)
C7	0.0763 (19)	0.0634 (17)	0.0583 (19)	0.0160 (15)	−0.0215 (15)	−0.0017 (14)
C8	0.0493 (15)	0.0527 (15)	0.0471 (16)	0.0037 (13)	0.0046 (12)	0.0088 (12)
C9	0.061 (2)	0.103 (3)	0.100 (3)	0.0384 (19)	0.0100 (18)	0.008 (2)
C10	0.0468 (19)	0.139 (4)	0.196 (6)	0.024 (2)	0.007 (2)	0.025 (4)
C11	0.0652 (17)	0.0544 (16)	0.0607 (19)	0.0094 (13)	0.0206 (14)	0.0102 (13)
C12	0.074 (2)	0.0620 (19)	0.091 (3)	−0.0053 (16)	0.0256 (19)	0.0133 (17)
C13	0.083 (2)	0.088 (2)	0.110 (3)	−0.023 (2)	0.007 (2)	−0.003 (2)
C14	0.102 (2)	0.081 (2)	0.056 (2)	0.008 (2)	0.0207 (18)	0.0155 (17)
C15	0.135 (4)	0.099 (3)	0.071 (3)	0.005 (2)	0.001 (2)	−0.009 (2)

### Geometric parameters (Å, º)

**Table:**

S1—O2	1.4183 (19)	C12—C13	1.504 (5)
S1—O3	1.4097 (19)	C14—C15	1.503 (5)
S1—N3	1.678 (2)	C3—H3A	0.9700
S1—N4	1.589 (2)	C3—H3B	0.9700
O1—N2	1.413 (3)	C4—H4	0.9300
O1—C1	1.343 (3)	C7—H7A	0.9600
O4—C8	1.205 (3)	C7—H7B	0.9600
O5—C8	1.327 (3)	C7—H7C	0.9600
O5—C9	1.458 (3)	C9—H9A	0.9700
N1—C1	1.283 (3)	C9—H9B	0.9700
N1—C2	1.373 (3)	C10—H10A	0.9600
N2—C2	1.291 (3)	C10—H10B	0.9600
N3—C3	1.476 (3)	C10—H10C	0.9600
N3—C4	1.344 (3)	C11—H11	0.9800
N4—C6	1.313 (3)	C12—H12A	0.9700
C1—C11	1.492 (4)	C12—H12B	0.9700
C2—C3	1.494 (3)	C13—H13A	0.9600
C4—C5	1.361 (3)	C13—H13B	0.9600
C5—C6	1.425 (3)	C13—H13C	0.9600
C5—C8	1.487 (3)	C14—H14A	0.9700
C6—C7	1.497 (3)	C14—H14B	0.9700
C9—C10	1.439 (5)	C15—H15A	0.9600
C11—C12	1.539 (4)	C15—H15B	0.9600
C11—C14	1.518 (4)	C15—H15C	0.9600
			
O2—S1—N3	107.26 (11)	N2—C2—C3	120.9 (2)
O2—S1—N4	110.56 (11)	N3—C3—C2	110.41 (18)
O3—S1—O2	116.64 (13)	N3—C4—C5	124.0 (2)
O3—S1—N3	106.67 (11)	C4—C5—C6	119.7 (2)
O3—S1—N4	111.17 (13)	C4—C5—C8	114.5 (2)
N4—S1—N3	103.55 (10)	C6—C5—C8	125.5 (2)
C1—O1—N2	106.41 (17)	N4—C6—C5	122.6 (2)
C8—O5—C9	116.2 (2)	N4—C6—C7	114.7 (2)
C1—N1—C2	102.8 (2)	C5—C6—C7	122.6 (2)
C2—N2—O1	102.71 (18)	O4—C8—O5	124.6 (2)
C3—N3—S1	118.03 (15)	O4—C8—C5	123.6 (2)
C4—N3—S1	118.60 (16)	O5—C8—C5	111.6 (2)
C4—N3—C3	121.5 (2)	C10—C9—O5	108.1 (3)
C6—N4—S1	122.23 (18)	C1—C11—C12	109.3 (2)
O1—C1—C11	116.3 (2)	C1—C11—C14	111.5 (2)
N1—C1—O1	112.9 (2)	C14—C11—C12	112.0 (3)
N1—C1—C11	130.7 (2)	C13—C12—C11	114.8 (3)
N1—C2—C3	123.9 (2)	C15—C14—C11	114.4 (3)
N2—C2—N1	115.1 (2)		
			
S1—N3—C3—C2	−71.7 (2)	N4—S1—N3—C4	31.0 (2)
S1—N3—C4—C5	−14.3 (3)	C1—O1—N2—C2	0.6 (3)
S1—N4—C6—C5	13.9 (3)	C1—N1—C2—N2	−0.9 (3)
S1—N4—C6—C7	−171.32 (19)	C1—N1—C2—C3	176.3 (2)
O1—N2—C2—N1	0.2 (3)	C1—C11—C12—C13	−62.4 (3)
O1—N2—C2—C3	−177.1 (2)	C1—C11—C14—C15	61.6 (4)
O1—C1—C11—C12	−66.6 (3)	C2—N1—C1—O1	1.4 (3)
O1—C1—C11—C14	57.8 (3)	C2—N1—C1—C11	−175.8 (3)
O2—S1—N3—C3	78.61 (19)	C3—N3—C4—C5	−178.3 (2)
O2—S1—N3—C4	−85.95 (19)	C4—N3—C3—C2	92.4 (3)
O2—S1—N4—C6	83.5 (2)	C4—C5—C6—N4	9.6 (4)
O3—S1—N3—C3	−47.1 (2)	C4—C5—C6—C7	−164.7 (2)
O3—S1—N3—C4	148.37 (19)	C4—C5—C8—O4	23.7 (4)
O3—S1—N4—C6	−145.3 (2)	C4—C5—C8—O5	−152.6 (2)
N1—C1—C11—C12	110.4 (3)	C6—C5—C8—O4	−149.2 (3)
N1—C1—C11—C14	−125.2 (3)	C6—C5—C8—O5	34.4 (3)
N1—C2—C3—N3	−50.0 (3)	C8—O5—C9—C10	−166.0 (3)
N2—O1—C1—N1	−1.3 (3)	C8—C5—C6—N4	−177.8 (2)
N2—O1—C1—C11	176.3 (2)	C8—C5—C6—C7	7.9 (4)
N2—C2—C3—N3	127.1 (2)	C9—O5—C8—O4	2.1 (4)
N3—S1—N4—C6	−31.1 (2)	C9—O5—C8—C5	178.4 (2)
N3—C4—C5—C6	−8.5 (4)	C12—C11—C14—C15	−175.5 (3)
N3—C4—C5—C8	178.1 (2)	C14—C11—C12—C13	173.4 (3)
N4—S1—N3—C3	−164.45 (17)		

### Hydrogen-bond geometry (Å, º)

**Table:**

*D*—H···*A*	*D*—H	H···*A*	*D*···*A*	*D*—H···*A*
C4—H4···N2^i^	0.93	2.54	3.431 (3)	161

Symmetry code: (i) −*x*+3/2, *y*+1/2, *z*.
